# DLK1 Is a Somato-Dendritic Protein Expressed in Hypothalamic Arginine-Vasopressin and Oxytocin Neurons

**DOI:** 10.1371/journal.pone.0036134

**Published:** 2012-04-26

**Authors:** Carine Villanueva, Sandrine Jacquier, Nicolas de Roux

**Affiliations:** 1 INSERM, U676, Paris, France; 2 Université Paris Diderot, Sorbonne Paris Cité, UMR676, Paris, France; Montana State University, United States of America

## Abstract

Delta-Like 1 Homolog, *Dlk1*, is a paternally imprinted gene encoding a transmembrane protein involved in the differentiation of several cell types. After birth, *Dlk1* expression decreases substantially in all tissues except endocrine glands. *Dlk1* deletion in mice results in pre-natal and post-natal growth deficiency, mild obesity, facial abnormalities, and abnormal skeletal development, suggesting involvement of *Dlk1* in perinatal survival, normal growth and homeostasis of fat deposition. A neuroendocrine function has also been suggested for DLK1 but never characterised. To evaluate the neuroendocrine function of DLK1, we first characterised *Dlk1* expression in mouse hypothalamus and then studied post-natal variations of the hypothalamic expression. Western Blot analysis of adult mouse hypothalamus protein extracts showed that *Dlk1* was expressed almost exclusively as a soluble protein produced by cleavage of the extracellular domain. Immunohistochemistry showed neuronal DLK1 expression in the suprachiasmatic (SCN), supraoptic (SON), paraventricular (PVN), arcuate (ARC), dorsomedial (DMN) and lateral hypothalamic (LH) nuclei. DLK1 was expressed in the dendrites and perikarya of arginine-vasopressin neurons in PVN, SCN and SON and in oxytocin neurons in PVN and SON. These findings suggest a role for DLK1 in the post-natal development of hypothalamic functions, most notably those regulated by the arginine-vasopressin and oxytocin systems.

## Introduction

Delta-Like 1 Homolog (DLK1), also known as preadipocyte factor 1 (Pref-1), is a transmembrane protein expressed at the cell surface. It comprises an extracellular domain containing epidermal growth factor (EGF)-like repeats, a transmembrane domain, and a short intracellular tail. DLK1 is encoded by a paternally imprinted gene located on chromosome 12 in mouse and chromosome 14 in human. In mouse, *Dlk1* is widely expressed in embryonic tissues, and its expression level decreases markedly after birth except in a few endocrine glands and a subset of dopaminergic neurons [1]–[3]. The protein shares structural characteristics with the Notch/Delta/Serrate family but lacks the DSL (Delta/Serrate/LAG-2) domain conserved in all classic Notch ligands [4]. Soluble DLK1 is generated by shedding from the transmembrane domain of the extracellular domain cleaved by the ADAM17/TACE enzyme [5]–[8].

In recent years, evidence has accumulated that DLK1 inhibits adipocyte differentiation [8], [9]. It is also involved in other biological processes such as determination of the fate of many cell types including pancreatic islet cells [10], myocytes [11], hepatocytes [12] and neurons [13]. In adults, *Dlk1* is expressed in the normal pituitary gland, spinal cord, pancreatic islet cells, adrenals, and leydig cells, strongly suggesting a role in endocrine-related functions. DLK1 has been demonstrated to suppress growth hormone expression in GH3 cells [14].

Mice lacking paternally expressed *Dlk1* display pre- and post-natal growth deficiency, obesity, facial abnormalities, and abnormal skeletal development. This phenotype is not observed after maternal transmission of the null allele [15]. Mice with double or triple doses of *Dlk1* display embryonic growth enhancement followed by a failure to thrive and peri-natal lethality [16]. These phenotypes resemble those seen in maternal or paternal unidisomy of chromosome 12 [16]. A similar phenotype associated with precocious puberty has been reported in patients with maternal uniparental disomy of the orthologous region of chromosome 14 (14q32) and potentially ascribed to absence of *Dlk1* expression [17].

Although there is some evidence that DLK1 may exert neuroendocrine effects [18]–[20], the hypothalamic functions of DLK1 have not been evaluated. The objective of this study was to characterize *Dlk1* expression in the mouse hypothalamus after birth in order to clarify the potential neuroendocrine function of DLK1. For that purpose, hypothalamic and pituitary DLK1 expression was analyzed, hypothalamic nuclei and neurons expressing DLK1 were characterized in adult mice and post-natal variations in hypothalamic *Dlk1* expression were quantified.

## Materials and Methods

### Mice

Wild-type Swiss mice were supplied by Janvier (Le Genest Saint Isle, France) and housed in cages with free access to food and tap water, a 12-h light-dark cycle (8:00 am/8:00 pm) and constant temperature (21°C). Full details of the study have been approved by the Robert Debré research council review board; the approval number is 2010-13/676-008. All experiments were carried out in compliance with the ethical rules of our institution (National Institute for Health and Medical Research, INSERM) and with the recommendations in the National Research Council's Guide for the Care and Use of Laboratory Animals. Experiments were conducted in male mice on post-natal day P6 (neonates), P20 (juveniles), and P60 (adults).

### Tissue dissection

On the day of sacrifice, the mice were weighed, anaesthetized with inhaled isoflurane, and killed by decapitation. The brains were removed and the hypothalami and pituitaries were dissected and frozen in isopentane dry ice at −40°C then stored at −80°C until RNA extraction. To assess possible changes in hypothalamic gene expression associated with puberty in male mice, animals were killed at three different stages of development (P6, P20, and P60).

### Immunohistochemistry

#### Tissue preparation

The mice were deeply anaesthetized with isoflurane and perfused intracardially for 2 to 5 min with 4% paraformaldehyde in 0.12 M phosphate buffer (PB), pH 7.4. After the perfusion, the brain was immediately removed and maintained in the same paraformaldehyde solution for approximately 3 hours, at 4°C. All specimens were then cryoprotected in 15% sucrose/PB for 24 hours at 4°C then in 30% sucrose/PB for 24 hours at 4°C, for preparation of 30-µm free-floating sections. The brains were frozen in liquid isopentane at −70°C and stored at −80°C until sectioning. Coronal sections 30 µm in thickness were cut on a cryostat and collected in PB.

#### Immunohistochemistry

For fluorescent staining, free-floating slices were incubated with 5% donkey serum in 0.3% Triton X-100 phosphate buffered saline (PBS) for 45 minutes, to block non-specific antibody reactions. The slices were then incubated overnight with primary antibodies at 4°C in 1% donkey serum in 0.3% Triton X-100 PBS. After several rinses with PBS, the slices were incubated with a secondary fluorescent antibody in 1% donkey serum in 0.3% Triton X-100 PBS for 2 hours at room temperature. After three washes in PBS, the sections were stained with TO-PRO-3 (1/500, Invitrogen), rinsed in PBS, collected on Superfrost Plus slides (Microm Microtech, Francheville, France), and covered with fluoromount (Southernbiotech, Birmingham, AL, USA) for fluorescence microscopy.

For peroxidase staining, free-floating slices were incubated with 5% donkey serum in 0.3% Triton PBS for 1 hour, then with primary antibody in 1% donkey serum in 0.3% Triton PBS overnight at 4°C. After several washes with PBS, the slices were incubated with the secondary biotinylated antibody in 1% donkey serum in 0.3% Triton PBS for 90 minutes at room temperature. After incubation with H_2_O_2_ 0.3% in PBS for 10 minutes, the slices were rinsed and the universal immunolabelling system streptavidin-peroxidase kit (Vectastain ABC kit, Vector Laboratories, Burlingame, CA, USA) was used to develop the reaction. Cresyl violet 1% was used to counterstain nuclei of the brain sections, and the slides were then mounted with Pertex resin.

Controls for double-labeling included omission of the primary antibodies to test for non-specific binding of the secondary antibodies and incubation with one primary but both secondary antibodies to demonstrate the absence of cross labeling. Specificity for DLK1 immunostaining was tested by immunoadsorption of the antibody (1 µg/ml) with 10 µg/ml of DLK1 peptide for 60 minutes at room temperature followed by centrifugation at 4°C for 10 minutes at 15 000 *g*. The supernatant was then used for immunochemistry as described above.

The images were acquired using a Zeiss Axio Observer inverted microscope equipped with an LSM 5 Exciter confocal scanning system (Carl Zeiss, Jena, Germany). Excitation and emission filters were as follows: Cy3, lex = 548 nm and lem = 560/600 nm; TO-PRO-3, lex = 633 nm and lem = 650/695 nm; and Alexa 488, lex = 488 nm and lem = 505/545 nm. The level of each coronal section from the bregma was determined according to the mouse brain atlas [21].

#### Antibody characterization

Specific information on company, immunogen and antigen specificity of the antibodies used in this study can be found below.

The goat polyclonal C-19 and the rabbit polyclonal H-118 antibodies (Santa Cruz Biotechnology, Santa Cruz, CA, USA) were raised against a C-terminal peptide or a fragment corresponding to amino acids 266–383 of the human DLK1 respectively. The 266–383 region of the human DLK1 comprises the C-terminal end of the extracellular domain (37 residues) including one proteolytic site, the transmembrane domain and the entire intracellular domain. The specificity of C-19 and H-118 has been previously established in immunoblot of liver extracts by showing a band at the expected size for the full length DLK1 [22]. In addition, immunostaining with the C-19 has been completely blocked by preadsorption with the DLK1 antigenic peptide (sc-8624p, Santa Cruz Biotechnology, see results below). The H-118 antibody staining matched the C-19 antibody (see results) and the mapping of *Dlk1* mRNA expression in mouse brain (Allen Institute Brain Atlas) [21].

Two antibodies against the microtubule-associated protein 2 (MAP2A-2B) and SMI-31 were used to determine the subcellular location of DLK1. The monoclonal MAP-2 mouse antiserum (Chemicon Millipore, Temecula, CA; MAB3418) was prepared against the bovine brain microtubule protein, recognizing a double band (corresponding to MAP-2a and MAP-2b) of 300 kDa (manufacturer's technical information). This antibody labeled the normal somato-dendritic distribution of MAP-2 in adult rodent neurons [23]. The mouse monoclonal antibody anti-SMI-31 raised against phosphorylated neurofilament (Sternberger monoclonals, Covance, San Diego, CA) was originally raised against rat hypothalamus proteins [24] and was demonstrated to label phosphorylated neurofilament in rat brainstem [25]. Jones et al have previously reported its specificity in rodent brain study [26].

Several antibodies were used to determine DLK1-expressing hypothalamic neurons. Specificities of these antibodies were established by different methods. i) Vasopressin: the rabbit polyclonal anti-vasopressin (Immunostar, Hudson, WI, USA) was raised against synthetic arginine vasopressin (AVP) coupled to bovine thyroglobulin. Previous studies have shown that preadsorption with synthetic vasopressin peptide (10 µM) resulted in a complete loss of immunolabeling in the paraventricular nucleus of rodent hypothalamus [27]. ii) Oxytocin (OXT): the rabbit polyclonal anti-oxytocin serum (Chemicon Millipore, AB911) was produced against full-length oxytocin conjugated to thyroglobulin. Specificity of the antibody and cross reactivity to arginine vasopressin was established by immunoblot (Chemicon information) and by preadsorbing the antisera using the synthetic peptide antigen, which resulted in a complete loss of immunolabeling [28]. iii) Vasoactive intestinal peptide (VIP): a polyclonal antibody (INCSTAR, #20077) was raised against VIP conjugated to bovine thyroglobulin with carbodiimide. The specificity of the antibody has been previously established [29]. iv) Growth-hormone-releasing hormone (GHRH): a polyclonal antibody (a gift from C. Loudes, INSERM U894, Paris France) was raised in rabbit against the 25-amino-acid C-terminal part of the mouse GHRH sequence in which the 17-Tyr moiety was replaced by a Cys to allow conjugation to keyhole limpet hemocyanin as carrier [30]. The specificity of the antibody was established by the co-staining of endogenous GHRH with eGFP fluorescence in GHRH-eGFP transgenic mouse [30]. v) Neuropeptide Y (NPY): the NPY antibody (a gift from E. Grouzmann, Lausanne, Switzerland) is a mouse monoclonal antibody raised against the neuropeptide NPY [31]. The specificity of this antibody has been previously established in the hypothalamus [32]. vi) Alpha-melanocyte-stimulating hormone (α-MSH): the sheep polyclonal α-MSH antiserum was raised against α-MSH conjugated with bovine thyroglobulin (Chemicon Millipore, #AB5087). The specificity of the α-MSH antibody was established by the complete absence of staining after pre-immunoadsorption with the immunogenic peptide [33]. vii) Kisspeptin: this antibody was raised against a synthetic mouse peptide Kp10 (A gift from Alain Caraty, IFR 145, INRA, Nouzilly, France). Its specificity has been established by the absence of staining after preimmunoadsorption with the antigenic peptide [34] and in *Kiss1* deleted mouse. Additionally, no cross-reactivity with peptides of similar size and/or known to be related peptides (e.g. prolactin-releasing peptide, a peptide of the RFamide family) has been shown [35]. viii) Glial fibrillary acidic protein (GFAP): the polyclonal rabbit antibody was raised against GFAP isolated from bovine spinal cord (reference Z0334, Dako, USA). The GFAP antibody recognizes the well-known intermediate filament protein expressed by astrocytes, and detects a band of 51 kDa on western blots of rodent brain extracts [36]. The secondary antibodies were Cy3 donkey anti-goat (Jackson, Suffolk, UK), Alexa 488 donkey anti-mouse (Invitrogen, Carlsbad, CA, USA), Alexa 488 donkey anti-rabbit (Invitrogen), peroxidase-conjugated AffinityPure donkey anti-rabbit IgG (Jackson), and peroxidase-conjugated donkey anti-goat IgG (Jackson).

### Western blot analysis

Hypothalami and pituitaries from male mice were micro-dissected, snap-frozen in liquid isopentane at −70°C and stored at −80°C until further analysis. Tissue was homogenised in RIPA buffer (50 mM Tris-HCl pH 8, 0.1% SDS, 1% NP40, 150 mM NaCl, 0.5%Na deoxycholate, 1 mM PMSF, and protease inhibitors). For Western blot analysis of total protein extract, tissues in lysis buffer were centrifuged at 25 000 *g* at 4°C for 15 minutes and the supernatant was collected and stored at −80° until further use. For Western blot analysis of membrane protein extract, tissues homogenised in lysis buffer (10 mM Tris-HCl pH 7.4, 1 mM EDTA, protease inhibitor (Boehringer Mannheim, Reims, France), and 1 mM PMSF) were centrifuged at 200 *g* at 4°C for 5 minutes and the supernatant was then centrifuged at 25 000 *g* at 4°C for 30 minutes. The supernatant containing the cytoplasmic proteins (post-nuclear supernatant) was collected and stored at −80° until further use. The pellet was solubilised in 50 mM Tris pH 6.8, 150 mM NaCl, 10% glycerol, and 1% Triton, incubated for 60 minutes at 4°C on a wheel, and centrifuged at 25 000 *g* for 15 min at 4°C. The supernatant was collected and stored at −80°C. Protein concentrations were determined using the BCA protein assay kit (Pierce, Rockford, IL, USA). Protein extracts (20 µg per sample) were denatured with β-mercaptoethanol and 5× SDS Page in loading buffer at 96°C for 5 minutes. Proteins were separated on polyacrylamide gel (8% or 4–20% gradient gel) and transferred onto polyvinylidene difluoride membranes (ProBlot, ABI, Foster City, CA, USA). The membranes were rinsed with Tween 0.2% containing PBS, saturated with blocking solution (3% donkey serum) for 1 hour at room temperature, and incubated overnight at 4°C with primary antibodies in PBS, 0.2% Tween 20, and 0.5% donkey serum. After incubation with peroxidase-labelled secondary antibody, enhanced chemiluminescence (Immun-Star WesternC, Biorad, Marnes-la-Coquette, France) was used to detect the immunoreactive protein, (Chemidoc, Biorad, Marnes-la-Coquette, France). Equal loading and transfer of samples were confirmed according to the β–actin signal (mouse anti-ß-actin antibody from Sigma, Saint Quentin Fallavier, Fr).

### Reverse-transcription polymerase chain reaction (RT-PCR)

Total RNA was extracted from the hypothalamic and pituitary tissues using Trizol, (Invitrogen). Reverse transcription into first-strand cDNA of total RNA (800 ng) was achieved using random primers and the SuperScript® II Reverse Transcriptase Reagent Kit (Invitrogen). PCR was performed to amplify cDNA in PCR buffer (50 mM Tris pH 8, 50 mM KCl) with 0.2 µM of each primer (table S1), 0.2 mM of dNTP and 1 µL of TAQ polymerase (Redgoldstar, Eurogentec, Seraing, Be) for 35 cycles after initial denaturation at 95°C for 5 min. The reaction products were separated on 2% agarose gel and visualised using an ultraviolet apparatus.

### Quantitative polymerase chain reaction analysis

The ABI Prism 7300 HT Sequence Detection System (PE Applied Biosystems, Foster City, CA, USA) was used to perform real-time PCR (see table S1 for primer sequences). Each target was amplified individually and in duplicate. Relative expression was calculated using the comparative threshold cycle (CT) method. For each quantitative PCR analysis, technical validation was performed according to standard procedures. A dissociation curve was plotted to check that a single amplicon was generated. The size of each amplicon was assessed on agarose gel to confirm primer specificity. PCR primers were validated when the slope generated using different cDNA dilutions was between −2.9 and −3.3 and the correlation coefficient was between 0.95 and 1.

### Statistical analysis

We computed the mean mRNA levels in the replicates for each experimental repeat. Experimental groups were compared using ANOVA (Turkey's post hoc test). All data were described as mean (±SEM). All statistical analyses were performed using GraphPad Prism 5.0 (GraphPad Software, San Diego, CA, USA).

## Results

### Two *Dlk1* isoforms are expressed in the hypothalamus and pituitary

**Figure 1:**
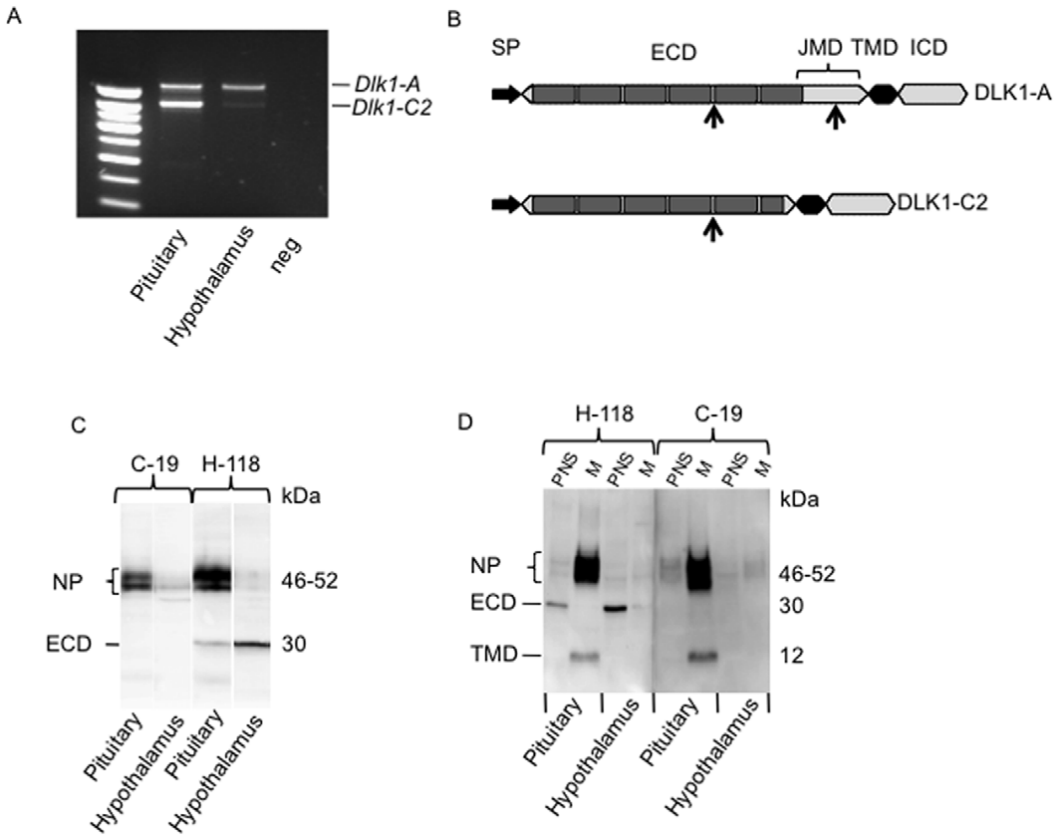
DLK1 expression in the adult male mouse hypothalamus and pituitary as uncleaved and cleaved proteins. (A) Reverse-transcription polymerase chain reaction (RT-PCR) was performed using two primers located in *Dlk1* exons 4 and 5 as described in materials and methods. (B) Schematic representation of the DLK1-A and C2 isoforms. The arrows indicate the proteolytic cleavage sites described by Smas et al. [5]. The dark grey boxes indicate epidermal growth factor (EGF)-like repeats. SP: signal peptide region. TMD: transmembrane domain. JMD: juxtamembrane domain. ECD: extracellular domain. ICD: intracellular domain. (C) Western blot on total proteins extracted from adult male mouse hypothalamus and pituitary. (D) Pituitary and hypothalamic DLK1 isoforms were characterized in post-nuclear supernatants (PNS) and membrane extracts (M). DLK1 isoforms were detected using a goat polyclonal anti-DLK1 antibody against the intracellular domain (C-19) and a rabbit polyclonal anti-DLK1 antibody against part of the extracellular domain, the transmembrane and the intracellular domain (H-118). NP, non-proteolysed DLK1.

To determine *Dlk1* isoforms expressed in the hypothalamus and pituitary, reverse transcribed total RNA was PCR amplified using two primers located in exons 4 and 5 (see table S1). Two PCR products of about 1000 bp and 800 bp were identified in both tissues (Figure 1A). After gel purification, sequencing showed that the two PCR products differed by a 225-bp deletion between nucleotides 836 and 1062 (cDNA numbering). These two mRNA isoforms have been described previously as isoform *Dlk1-A*, which is translated into the full-length protein (DLK1-A) and isoform *Dlk1-C2* arising by differential splicing within exon 5, which produces a shorter protein chain (DLK1-C2) [37]. The relative expression of the two *Dlk1* mRNA isoforms differed between the hypothalamus and pituitary: *Dlk1-A* was expressed at a higher level than *Dlk1-C2* in the hypothalamus, whereas *Dlk1-C2* predominated in the pituitary (Figure 1A).

DLK1-A and DLK1-C2 differed by 75 residues in the juxtamembrane region (Figure 1B). A cleavage site has been described in this juxtamembrane region of the extracellular domain of DLK1-A but not DLK1-C2 [5]. In total protein extracts from both the pituitary and the hypothalamus, an antibody raised against the 266–383 region of human DLK1 (H-118) which comprises part of the extra-cellular domain (24–303), the transmembrane domain (304–327) and the intra-cellular domain (328–383), detected three bands, of 52-kDa, 46-kDa and 30-kDa, respectively. The C-19 antibody directed against a C-terminal peptide of the intracellular domain revealed very faint bands at 46-kDa and at 52-kDa but no band at 30-kDa (Figure 1C). We suspected that the 46-kDa and 52-kDa bands corresponded to uncleaved glycosylated forms of DLK1 and that the 30-kDa band corresponded to a soluble form of DLK1 probably cleaved from the DLK1-A. This DLK1soluble form was missing the intracellular domain. To confirm this result, we compared DLK1 protein isoforms from post-nuclear supernatants to molecular forms solubilized from membrane extracts (Figure 1D). 46- and 52-kDa bands were very intense in membrane extracts of pituitary (Figure 1D) whereas the intensity of these two bands was very low in membrane extracts of hypothalamus (Figure 1D). The 30-kDa molecular form was found in post-nuclear supernatants of both tissues but not in membrane extracts (Figure 1D). In the pituitary, the 30-kDa band was less intense than the 46- and 52-kDa bands. In contrast, the 30-kDa band in the hypothalamus was almost the only visible band.

Both antibodies identified an additional band, of 12 kDa, in pituitary membrane extracts (Figure 1D). We assumed that this 12-kDa band corresponded to a protein fragment composed of parts of the transmembrane and intracellular DLK1 domains.

Thus, DLK1 was expressed in pituitary and hypothalamic tissue as an uncleaved transmembrane protein, and a soluble 30-kDa isoform composed only of the extracellular domain. A small 12-kDa transmembrane protein probably containing the intracellular domain was only found in the pituitary. Pituitary tissue chiefly expressed uncleaved DLK1, whereas the 30-kDa soluble DLK1 was the predominant form in adult hypothalamic tissue.

### DLK1 is expressed in the arcuate, paraventricular, supraoptic, suprachiasmatic, dorsomedial and lateral hypothalamic nuclei

**Figure 2:**
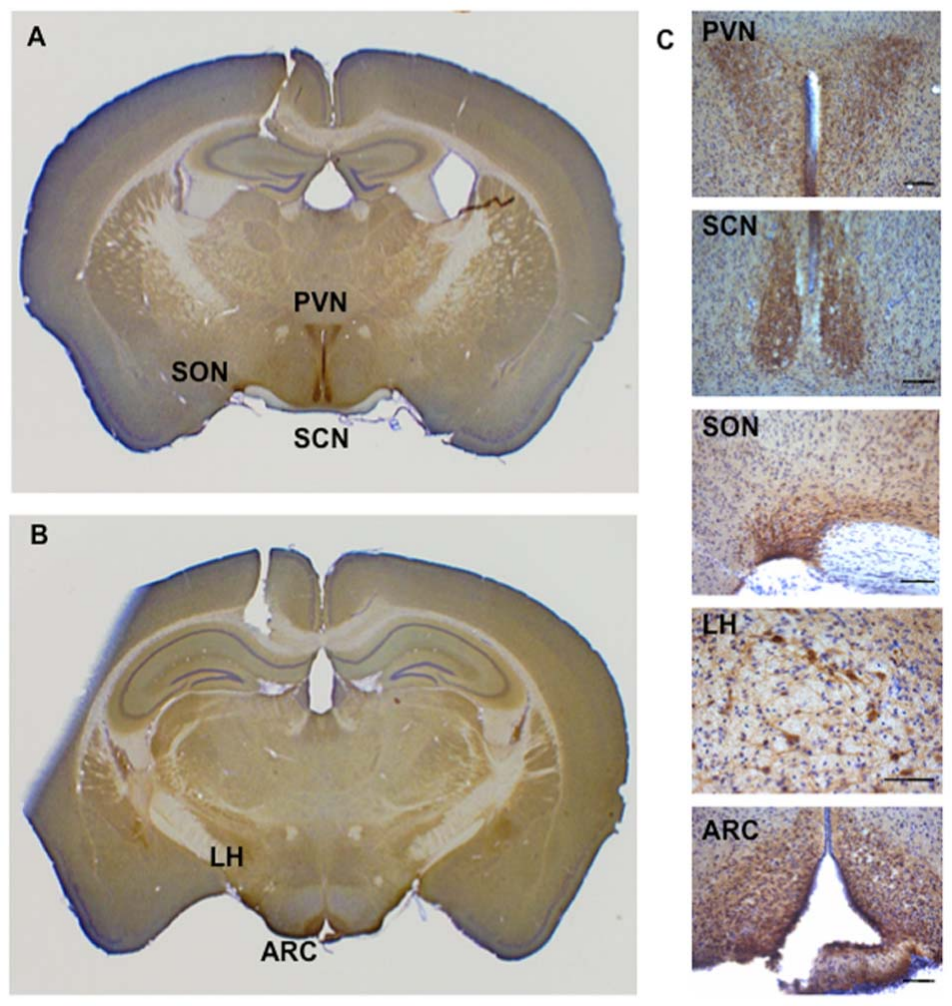
DLK1 is expressed in specific nuclei of the hypothalamus. **A**) Immunohistochemistry using the C-19 antibody was performed on free-floating coronal brain sections from adult male mice as described in materials and methods (2,5× objective; Bregma −0.82 mm at the hypothalamic level according to the mouse brain atlas [21]. B) Bregma −2.06 mm C) Higher magnification of the paraventricular nucleus (PVN), suprachiasmatic nucleus (SCN), supraoptic nucleus (SON) arcuate nucleus (ARC), and lateral hypothalamic nucleus (LH) (20× objective; scale bar: 80 µm).

Immunohistochemistry (IHC) performed using the C-19 antibody showed intense labeling in the suprachiasmatic nucleus (SCN) and weaker labeling in the paraventricular nucleus (PVN), supraoptic nucleus (SON), arcuate nucleus (ARC), dorsomedial (DMN) and lateral hypothalamic nucleus (LH) (Figure 2). The same staining was found with the H-118 antibody produced in rabbit and directed against a region of the protein comprising parts of extracellular and intracellular domains of DLK1 (data not shown). The absence of staining after immunoabsorption of C-19 antibody by the DLK1 antigenic peptide as well as DLK1 staining in somatotrophs in the anterior pituitary (figure S1A) show that C-19 hypothalamic staining was specific [38]. Furthermore, this DLK1 staining reproduced the pattern observed by in-situ hybridization (http://mouse.brain-map.org/gene/show/13165). Indeed with a probe against nucleotides 426 to 1229 (NM_010052), a signal was observed in the PVN, SCN, SON, DMN and ARC of the mouse hypothalamus.

**Figure 3:**
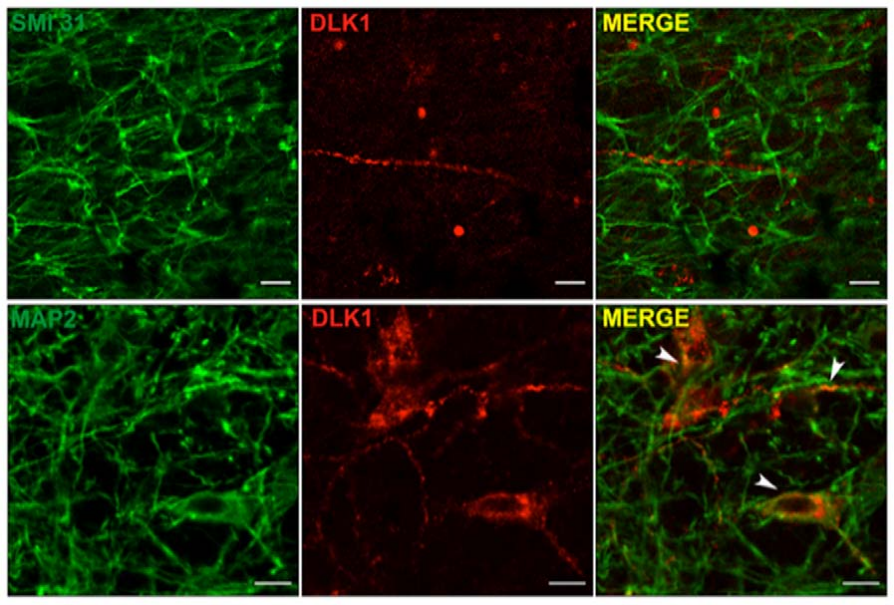
DLK1 expression is restricted to dendrites and perikarya of hypothalamic neurons. Dual immunofluorescence staining for DLK1 (C-19) and phosphorylated neurofilament (SMI-31) or microtubule-associated protein 2 (MAP-2) on free-floating sections of hypothalami from adult male mice. Tissue was fixed and processed as described in materials and methods. Arrowheads indicate co-staining of DLK1 and MAP-2 in dendrites and perykarya. DLK1 do not colocalize with SMI-31 (40× objective for MAP-2 and 63× objective for SMI-31, scale bar: 10 µm).

To specify the subcellular location of DLK1, we performed dual IHC with antibodies against DLK1 and specific markers of dendrites or axons. MAP-2 is involved in microtubule assembly and is found in large amounts in dendrites. Double IHC with an antibody raised against MAP-2 showed co-staining with DLK1 in dendrites as well as in perikarya (Figure 3), whereas DLK1 was never co-stained with SMI-31, a protein active primarily in the distal portions of axons (Figure 3). No co-staining was found with the glial fibrillary acidic protein (GFAP) (data not shown).

DLK1 thus emerged as a somatodendritic protein expressed in neurons located in the PVN, SCN, SON, DMN, ARC and LH nuclei.

### Hypothalamic *Dlk1* expression increases between birth and adulthood

**Figure 4:**
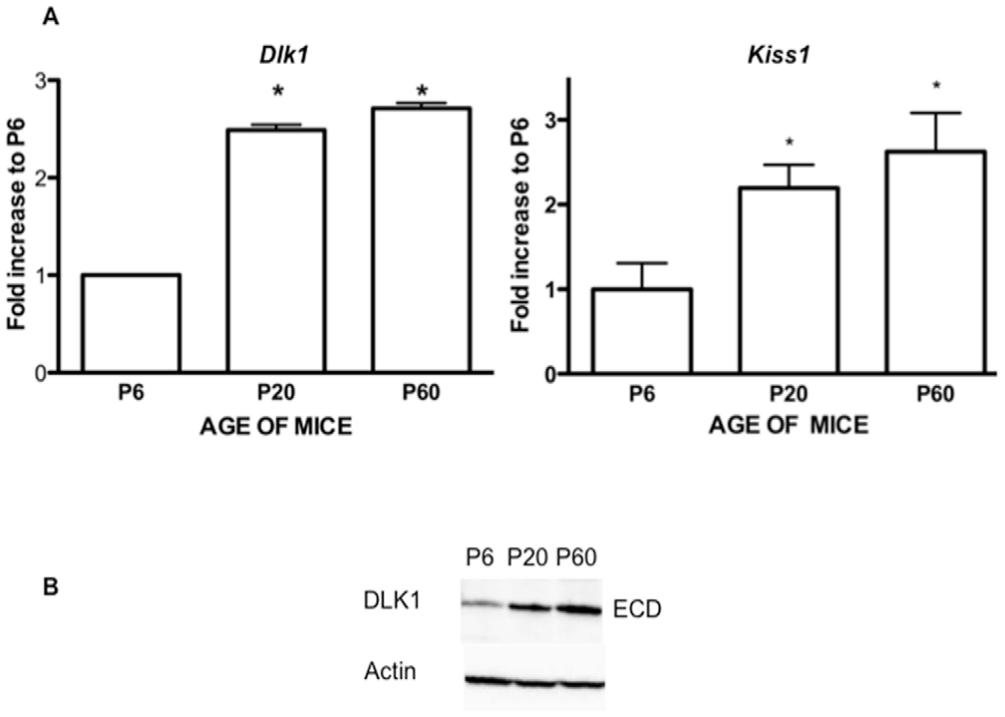
*Dlk1* expression increases in the hypothalamus between birth and adulthood. A) Total hypothalamic RNA was extracted on post-natal days 6, 20 and 60 (P6, P20, P60). mRNA levels were quantified using real-time RT-PCR as described in the materials and methods section. The graphs show fold increases in expression at P20 and P60 compared to P6. *Kiss1* expression was quantified using real-time RT-PCR as described in the materials and methods section. The graphs represent pooled data (mean ± SEM) from three different experiments with 4 mice per group. *indicates a significant increase (*p*<0.01) relative to P6. B) DLK1 protein levels on P6, P20 and P60 were evaluated using western blot on total cellular extracts with the H-118 antibody.

As persistent *Dlk1* expression was found in the adult mouse hypothalamus, it was of interest to analyse variations in hypothalamic *Dlk1* expression after birth. Significant increases in *Dlk1* mRNA were observed between P6 and P20 (2.40±0.14-fold increase (FI), n = 4, *p*<0.01) and between P6 and P60 (2.80±0.15 FI, n = 4, *p*<0.01) (Figure 4A). These increases were similar to that seen for *Kiss1* expression (P6 vs. P20, 2.20±0.27 FI; n = 4, *p*<0.05; P6 vs. P60, 2.6±0.46 FI; n = 4, *p*<0.01) (Figure 4A), which was recently described as a major determinant of pubertal onset [39], [40]. Western Blot analysis of total extracts of P6, P20, and P60 hypothalamic tissues from male mice showed that the 30-kDa isoform levels increased from P6 to P20 and P60 (Figure 4B). The post-natal increase in hypothalamic *Dlk1* mRNA was therefore associated with increased expression of the protein. DLK1 was expressed in the same nuclei (ARC, PVN, SCN, DMN, SON and LH) at all three post-natal time points (P6, P20, and P60) (see figure S2), indicating that the post-natal increase in hypothalamic DLK1 expression resulted from modulation of existing expression and not from expression by additional hypothalamic nuclei.

### *Dlk1* is expressed in arginine-vasopressin and oxytocin neurons

**Figure 5:**
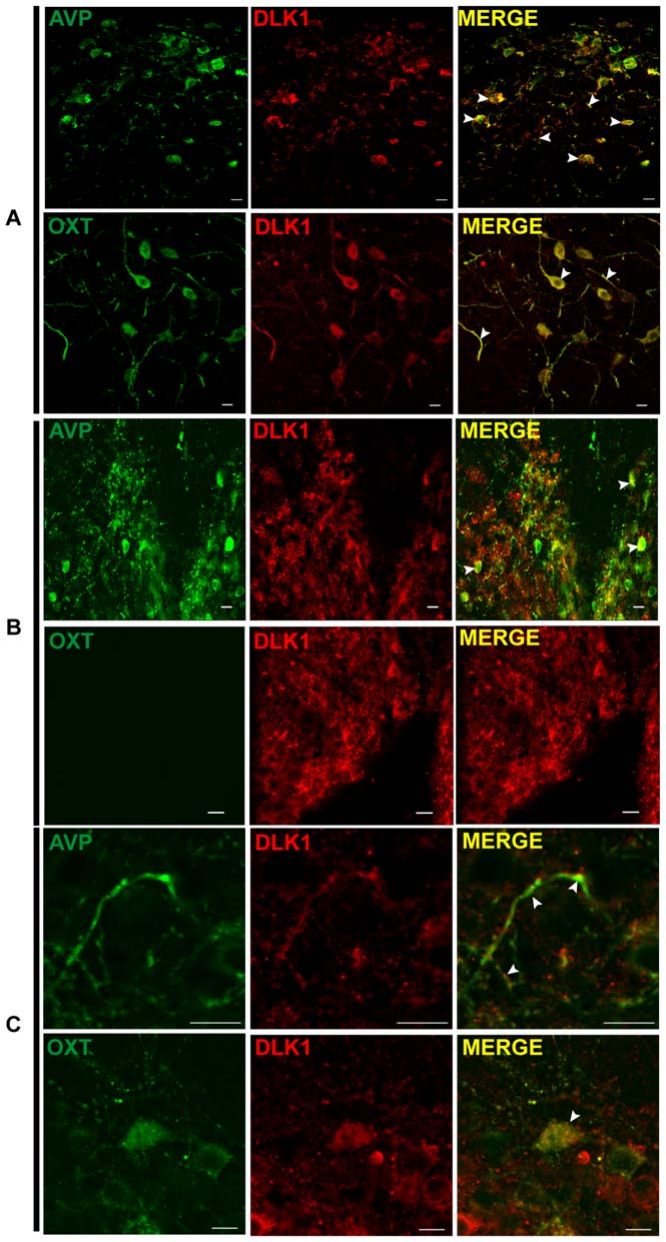
DLK1 expression in AVP and OXT neurons in the PVN, the SCN and the SON. Dual immunofluorescence staining of DLK1 (antibody C-19) and AVP or OXT on 0.6 µm-thick confocal PVN (A), SCN (B) and SON (C) sections obtained using a 63× objective. Arrowheads indicate double staining. Scale bars: 10 µm.

Our finding of DLK1 expression in the SCN, PVN and SON suggested expression in neurons expressing vasoactive intestinal peptide, arginine-vasopressin or oxytocin. Double IHC with the C-19 antibody and a rabbit antibody against VIP revealed that VIP-expressing neurons did not express DLK1 (data not shown). Double IHC with an antibody against AVP or OXT showed DLK1 expression in AVP and OXT neurons in the PVN (Figure 5A). In this nucleus, very few AVP+ neurons do not express DLK1 whereas in the LH, the majority of DLK1+ neurons do not express AVP or OXT (data not shown). Co-immunostaining between DLK1 and AVP was also observed in the SCN and as expected, there were no OXT+ neurons in this nucleus (Figure 5B). In the SON, AVP+ neurons express DLK1, but few DLK1+ fibers were not stained for AVP (Figure 5C). In this nucleus, DLK1 immunostaining was observed in OXT+ perikarya and fibers (Figure 5C). Double IHC with antibodies against GHRH, NPY, α-MSH, and Kisspeptin showed no co-staining with DLK1 in the ARC (data not shown). There was no evidence of DLK1 immunostaining in AVP axonal extremities in the neuronal lobe of the pituitary (Figure S1B).

Altogether, these results indicated that AVP-expressing neurons co-express DLK1 in the PVN, SCN, and SON. OXT-expressing neurons co-express DLK1 in the PVN and the SON mostly in the neurons fibers. Few DLK1-expressing neurons in the LH express AVP or OXT.

## Discussion

Very little is known about the biological function of Dlk1. Expression of Dlk1 is found in many embryonic tissues but decreases in several tissues after birth, an event that is critical to various processes leading to cell differentiation [1], [2]. A neuroendocrine function has been hypothesized for DLK1 but has never been characterized. Our results show that DLK1 expression in the hypothalamus occurs mainly as a cleaved protein and it is expressed in dendrites of AVP and OXT neurons in the PVN and LH nuclei. In the SON, DLK1 was co-stained mostly with AVP and only with few OXT-expressing neurons. DLK1 expression was also found in neurons negatives for AVP, OXT and other neurons negatives for peptides expressed in the ARC nucleus such as NPY, α–MSH, GHRH, Kisspeptin. The hypothalamic *Dlk1* expression increases after birth suggesting that DLK1 expression is associated with the post-natal maturation of hypothalamic neurons.

DLK1-A and DLK1-C2 are transmembrane proteins of 385 and 310 amino acids with calculated molecular weights at 41-kDa and 33-kDa, respectively. DLK1-A differs from the DLK1-C2 by the presence of a sequence of 75 amino acids bearing a proteolytic cleavage site. The mouse hypothalamus mainly expresses a soluble 30-kDa DLK1 protein fragment. This DLK1 protein fragment is recognized by an antibody directed against the extracellular domain but not by an antibody directed against the intracellular domain of DLK1. From this information, we can hypothesize that the 30-kDa protein fragment is a part of the extracellular domain of DLK1. Furthermore, the mouse hypothalamus mainly expresses the *Dlk1-A* mRNA isoform. From this, we propose that the hypothalamic DLK1 is mainly expressed as a cleaved DLK1-A isoform. However, the expression of DLK1 in the pituitary differs from that of the hypothalamus. The pituitary expresses a high amount of two 46-kDa and 52-kDa uncleaved DLK1 and low amount of 30-kDa and 12-kDa DLK1 protein fragments. Important, the pituitary expresses higher amount of *Dlk1-C2* mRNA than *Dlk1-A* mRNA and *Dlk1-C2* encodes for a protein lacking the extracellular domain cleavage site present in DLK1-A. We therefore propose that the 46- and 52-kDa DLK1 molecular forms expressed in the pituitary are two different O- or N-glycosylation states of the DLK1-C2 isoform. The difference between DLK1 molecular weights found in our study and in earlier studies [8] may reflect differences in the O- or N-glycosylation status of the extracellular domain in neurons compared to COS cells or fibroblasts [41]. Further analysis of the glycosylation status of DLK1 is required to confirm this tissue-specific post-translational modification. The 12-kDa-protein fragment was found in pituitary but not in hypothalamic membrane extracts. Therefore, this 12-kDa fragment is probably cleaved from the DLK1-C2 isoform.

The hypothalamus mainly expressed cleaved DLK1, whereas the pituitary expressed mainly uncleaved DLK1. A larger amount of *Dlk1-C2* mRNA than of *Dlk1-A* mRNA is expressed in the pituitary, which contrasts with the marked predominance of *Dlk1-A* mRNA in the hypothalamus. The DLK1-C2 isoform lacks the juxtamembrane cleavage site recognized by the ADAM enzyme involved in one of the cleavages of the DLK1 extracellular domain [8]. Further work is needed to determine whether differences in the relative expression levels of DLK1-A and DLK1-C2 between the hypothalamus and pituitary may serve to regulate the ratio of cleaved to uncleaved DLK1, and therefore the biochemical function of this protein.

The high level of DLK1 expression in the SCN is striking and co-staining was found with AVP expressing neurons. In the SON, DLK1 is mainly expressed in AVP-expressing neurons. The co-staining of DLK1 with MAP-2 and AVP or OXT-expressing neurons was associated with the absence of DLK1 staining in the neural lobe of the pituitary, where AVP and OXT axons terminate. DLK1 is thus a somatodendritic protein of AVP- and OXT-expressing neurons. AVP and OXT are two neuropeptides secreted from two types of neurons, namely, the magnocellular and parvocellular neurons. The AVP from magnocellular neurons from the PVN and the SON acts as a neurohormone. It is transported through the axon passes through the inner zone of the median eminence, and reaches the nervous lobe of the pituitary gland; it is then secreted into the circulation to modulate the activity of certain organs such as kidney and therefore involved in osmoregulation. Peripheral actions of OXT are to stimulate uterine contraction and lactation.

Central dendritic release from magnocellular neurons have been described for both AVP and OXT peptides [42]. Central effects of both neuropeptides, AVP and OXT have been well documented in the regulation of social behavior, circadian rhythm as well as modulation of the hypothalamic-pituitary adrenals axis in response to stress [42], [43]. As dendritic arborescence of AVP neurons changes between birth and weaning [44], it would be interesting to determine whether DLK1 is involved in the post-natal plasticity of AVP neurons.

We observed that variations in hypothalamic *Dlk1* expression between the early post-natal period and adulthood in mice correlate negatively with global variations in the expression of the Notch target gene Hes5 and the Notch ligand Jagged2 (see table S2). As notch pathway is actively involved in neurogenesis, axon and dendrite growth and synaptic plasticity [45]–[47], further work is necessary to determine whether DLK1 could play a role in the post-natal maturation of hypothalamic neurons via the Notch pathway as described for adipocyte differentiation [48]–[50]. A trans effect of DLK1 on the Notch pathway was proposed [3], [51] as well as a cis-inhibitory effect which was more pronounced with the uncleaved form than with the cleaved form [49]. However, due to the absence of valuable antibodies against Notch pathway actors for IHC, we were unable to determine whether DLK1 could acts in cis or in trans with the Notch pathway in the hypothalamus. An effect of DLK1 on hypothalamic neurons via integrin signalling and SOX9 upregulation may be another hypothesis [52].

Very recently, Ferron et al described a role for Dlk1 in neurogenesis within the sub-ventricular zone initiating early in postnatal life [53]. They showed that DLK1 maintains the potential of cell renewal from a pool of neural stem cells in coordination with niche astrocytes. The hypothalamus is also the place of an active neurogenesis in adulthood starting early in postnatal life [54] and having a potential role in energy balance [55]. Although, DLK1 staining in the hypothalamus looks broader than the staining observed with a cell-proliferation marker [55], it will be interesting to test whether DLK1 is involved in the regulation of the postnatal neurogenesis in the hypothalamus.

In summary, the data presented here show marked hypothalamic expression of DLK1 in the ARC, DMN, PVN, SON, SCN, and LH nuclei. DLK1 is expressed in arginine-vasopressin and in oxytocin expressing neurons but also in neurons that remain to be characterized in the ARC and DMN. *Dlk1* hypothalamic expression increases between birth and adulthood. We propose that DLK1 may have a dual role in the hypothalamus: it may play a role in the post-natal hypothalamic maturation of the AVP and OXT neurons. Further work is necessary to investigate whether Dlk1 is involved in the differentiation process of hypothalamic neuronal stem cells.

## Supporting Information

Figure S1 **Dlk1 pituitary immunostaining. A) Pituitary immunostaining with antibodies to DLK1 (C-19) and growth hormone (GH).** A confocal 0.6 µm-thick section obtained with a 63× objective. Primary antibody used for GH staining is the polyclonal guinea pig anti rat growth hormone (NIDDK, Torrance, CA). Arrow heads indicate double staining. **B**) **DLK1 is not expressed in the neuronal lobe of the pituitary.** Dual immunofluorescence staining on a single 0.6 µm-thick confocal section obtained using a 40× objective. Scale bars, 10 µm; AP: anterior pituitary; NL: neuronal lobe of the pituitary.
(TIF)

Figure S2 **Hypothalamic nuclei expressing DLK1 do not change with age in male mice.** Immunostaining with the C-19 antibody was performed on free-floating sections at P6, P20, and P60 as described in the “materials and methods" section. Confocal 0.6 µm-thick sections were obtained with a 40× objective. Suprachiasmatic nucleus (SCN), Paraventricular nucleus (PVN), lateral hypothalamic nucleus (LH), supraoptic nucleus (SON), and arcuate nucleus (ARC). Scale bars: 50 µm. The stained hypothalamic nuclei are the same at the different ages. To quantify a possible variation of DLK1 expression in each hypothalamic nucleus at the different stages, RT-qPCR of Dlk1 should be performed on dissected hypothalamic nuclei.
(TIF)

Table S1 **PCR primers.** Sequences of PCR primers used for polymerase-chain-reaction amplification (PCR) or quantitative real-time PCR (qPCR).
(DOC)

Table S2 **Hypothalamic mRNA expression of Notch pathway genes on post-natal days (P) 6, 20 and 60.** Total RNA from pooled hypothalami (n = 4) was reverse-transcribed and analysed using quantitative PCR as described in materials and methods. The mRNA levels are given relative to GAPDH mRNA levels. ns: not significant.
(DOC)

## References

[1] Sul HS (2009). Minireview: Pref-1: role in adipogenesis and mesenchymal cell fate.. Mol Endocrinol.

[2] Laborda J (2000). The role of the epidermal growth factor-like protein dlk in cell differentiation.. Histol Histopathol.

[3] Bauer M, Szulc J, Meyer M, Jensen CH, Terki TA (2008). Delta-like 1 participates in the specification of ventral midbrain progenitor derived dopaminergic neurons.. J Neurochem.

[4] Gordon WR, Arnett KL, Blacklow SC (2008). The molecular logic of Notch signaling–a structural and biochemical perspective.. J Cell Sci.

[5] Smas CM, Chen L, Sul HS (1997). Cleavage of membrane-associated pref-1 generates a soluble inhibitor of adipocyte differentiation.. Mol Cell Biol.

[6] Bachmann E, Krogh TN, Hojrup P, Skjodt K, Teisner B (1996). Mouse fetal antigen 1 (mFA1), the circulating gene product of mdlk, pref-1 and SCP-1: isolation, characterization and biology.. J Reprod Fertil.

[7] Jensen CH, Krogh TN, Hojrup P, Clausen PP, Skjodt K (1994). Protein structure of fetal antigen 1 (FA1). A novel circulating human epidermal-growth-factor-like protein expressed in neuroendocrine tumors and its relation to the gene products of dlk and pG2.. Eur J Biochem.

[8] Wang Y, Sul HS (2006). Ectodomain shedding of preadipocyte factor 1 (Pref-1) by tumor necrosis factor alpha converting enzyme (TACE) and inhibition of adipocyte differentiation.. Mol Cell Biol.

[9] Smas CM, Sul HS (1993). Pref-1, a protein containing EGF-like repeats, inhibits adipocyte differentiation.. Cell.

[10] Carlsson C, Tornehave D, Lindberg K, Galante P, Billestrup N (1997). Growth hormone and prolactin stimulate the expression of rat preadipocyte factor-1/delta-like protein in pancreatic islets: molecular cloning and expression pattern during development and growth of the endocrine pancreas.. Endocrinology.

[11] Crameri RM, Langberg H, Magnusson P, Jensen CH, Schroder HD (2004). Changes in satellite cells in human skeletal muscle after a single bout of high intensity exercise.. J Physiol.

[12] Tanimizu N, Tsujimura T, Takahide K, Kodama T, Nakamura K (2004). Expression of Dlk/Pref-1 defines a subpopulation in the oval cell compartment of rat liver.. Gene Expr Patterns.

[13] Costaglioli P, Come C, Knoll-Gellida A, Salles J, Cassagne C (2001). The homeotic protein dlk is expressed during peripheral nerve development.. FEBS Lett.

[14] Ansell PJ, Zhou Y, Schjeide BM, Kerner A, Zhao J (2007). Regulation of growth hormone expression by Delta-like protein 1 (Dlk1).. Mol Cell Endocrinol.

[15] Moon YS, Smas CM, Lee K, Villena JA, Kim KH (2002). Mice lacking paternally expressed Pref-1/Dlk1 display growth retardation and accelerated adiposity.. Mol Cell Biol.

[16] da Rocha ST, Charalambous M, Lin SP, Gutteridge I, Ito Y (2009). Gene dosage effects of the imprinted delta-like homologue 1 (dlk1/pref1) in development: implications for the evolution of imprinting.. PLoS Genet.

[17] Ogata T, Kagami M, Ferguson-Smith AC (2008). Molecular mechanisms regulating phenotypic outcome in paternal and maternal uniparental disomy for chromosome 14.. Epigenetics.

[18] Laborda J, Sausville EA, Hoffman T, Notario V (1993). dlk, a putative mammalian homeotic gene differentially expressed in small cell lung carcinoma and neuroendocrine tumor cell line.. J Biol Chem.

[19] Van Limpt VA, Chan AJ, Van Sluis PG, Caron HN, Van Noesel CJ (2003). High delta-like 1 expression in a subset of neuroblastoma cell lines corresponds to a differentiated chromaffin cell type.. Int J Cancer.

[20] Hsiao CC, Huang CC, Sheen JM, Tai MH, Chen CM (2005). Differential expression of delta-like gene and protein in neuroblastoma, ganglioneuroblastoma and ganglioneuroma.. Mod Pathol.

[21] Franklin KBJ, Paxinos G (2008). The Mouse Brain in stereotaxic coordinates.

[22] Huang J, Zhang X, Zhang M, Zhu JD, Zhang YL (2007). Up-regulation of DLK1 as an imprinted gene could contribute to human hepatocellular carcinoma.. Carcinogenesis.

[23] Blanchart A, De Carlos JA, Lopez-Mascaraque L (2006). Time frame of mitral cell development in the mice olfactory bulb.. The Journal of comparative neurology.

[24] Sternberger LA, Harwell LW, Sternberger NH (1982). Neurotypy: regional individuality in rat brain detected by immunocytochemistry with monoclonal antibodies.. Proceedings of the National Academy of Sciences of the United States of America.

[25] Sternberger LA, Sternberger NH (1983). Monoclonal antibodies distinguish phosphorylated and nonphosphorylated forms of neurofilaments in situ.. Proceedings of the National Academy of Sciences of the United States of America.

[26] Jones LG, Prins J, Park S, Walton JP, Luebke AE (2008). Lead exposure during development results in increased neurofilament phosphorylation, neuritic beading, and temporal processing deficits within the murine auditory brainstem.. The Journal of comparative neurology.

[27] McClellan KM, Stratton MS, Tobet SA (2010). Roles for gamma-aminobutyric acid in the development of the paraventricular nucleus of the hypothalamus.. The Journal of comparative neurology.

[28] Das M, Vihlen CS, Legradi G (2007). Hypothalamic and brainstem sources of pituitary adenylate cyclase-activating polypeptide nerve fibers innervating the hypothalamic paraventricular nucleus in the rat.. The Journal of comparative neurology.

[29] Cantwell EL, Cassone VM (2006). Chicken suprachiasmatic nuclei: II. Autoradiographic and immunohistochemical analysis.. The Journal of comparative neurology.

[30] Bouyer K, Loudes C, Robinson IC, Epelbaum J, Faivre-Bauman A (2007). Multiple co-localizations in arcuate GHRH-eGFP neurons in the mouse hypothalamus.. Journal of chemical neuroanatomy.

[31] Grouzmann E, Comoy E, Walker P, Burnier M, Bohuon C (1992). Production and characterization of four anti-neuropeptide Y monoclonal antibodies.. Hybridoma.

[32] Bergonzelli GE, Pralong FP, Glauser M, Cavadas C, Grouzmann E (2001). Interplay between galanin and leptin in the hypothalamic control of feeding via corticotropin-releasing hormone and neuropeptide Y.. Diabetes.

[33] Gallagher SK, Witkovsky P, Roux MJ, Low MJ, Otero-Corchon V (2010). beta-Endorphin expression in the mouse retina.. The Journal of comparative neurology.

[34] Richard N, Galmiche G, Corvaisier S, Caraty A, Kottler ML (2008). KiSS-1 and GPR54 genes are co-expressed in rat gonadotrophs and differentially regulated in vivo by oestradiol and gonadotrophin-releasing hormone.. Journal of neuroendocrinology.

[35] Franceschini I, Lomet D, Cateau M, Delsol G, Tillet Y (2006). Kisspeptin immunoreactive cells of the ovine preoptic area and arcuate nucleus co-express estrogen receptor alpha.. Neuroscience letters.

[36] Sultan-Styne K, Toledo R, Walker C, Kallkopf A, Ribak CE (2009). Long-term survival of olfactory sensory neurons after target depletion.. The Journal of comparative neurology.

[37] Smas CM, Green D, Sul HS (1994). Structural characterization and alternate splicing of the gene encoding the preadipocyte EGF-like protein pref-1.. Biochemistry.

[38] Larsen JB, Jensen CH, Schroder HD, Teisner B, Bjerre P (1996). Fetal antigen 1 and growth hormone in pituitary somatotroph cells.. Lancet.

[39] Seminara SB, Messager S, Chatzidaki EE, Thresher RR, Acierno JS (2003). The GPR54 gene as a regulator of puberty.. N Engl J Med.

[40] de Roux N, Genin E, Carel JC, Matsuda F, Chaussain JL (2003). Hypogonadotropic hypogonadism due to loss of function of the KiSS1-derived peptide receptor GPR54.. Proc Natl Acad Sci U S A.

[41] Krogh TN, Bachmann E, Teisner B, Skjodt K, Hojrup P (1997). Glycosylation analysis and protein structure determination of murine fetal antigen 1 (mFA1)–the circulating gene product of the delta-like protein (dlk), preadipocyte factor 1 (Pref-1) and stromal-cell-derived protein 1 (SCP-1) cDNAs.. Eur J Biochem.

[42] Landgraf R, Neumann ID (2004). Vasopressin and oxytocin release within the brain: a dynamic concept of multiple and variable modes of neuropeptide communication.. Front Neuroendocrinol.

[43] Ueta Y, Dayanithi G, Fujihara H (2011). Hypothalamic vasopressin response to stress and various physiological stimuli: visualization in transgenic animal models.. Horm Behav.

[44] Chevaleyre V, Moos FC, Desarmenien MG (2001). Correlation between electrophysiological and morphological characteristics during maturation of rat supraoptic neurons.. Eur J Neurosci.

[45] Redmond L, Oh SR, Hicks C, Weinmaster G, Ghosh A (2000). Nuclear Notch1 signaling and the regulation of dendritic development.. Nat Neurosci.

[46] Imayoshi I, Sakamoto M, Yamaguchi M, Mori K, Kageyama R (2010). Essential roles of Notch signaling in maintenance of neural stem cells in developing and adult brains.. J Neurosci.

[47] Breunig JJ, Silbereis J, Vaccarino FM, Sestan N, Rakic P (2007). Notch regulates cell fate and dendrite morphology of newborn neurons in the postnatal dentate gyrus.. Proc Natl Acad Sci U S A.

[48] Nueda ML, Baladron V, Sanchez-Solana B, Ballesteros MA, Laborda J (2007). The EGF-like protein dlk1 inhibits notch signaling and potentiates adipogenesis of mesenchymal cells.. J Mol Biol.

[49] Bray SJ, Takada S, Harrison E, Shen SC, Ferguson-Smith AC (2008). The atypical mammalian ligand Delta-like homologue 1 (Dlk1) can regulate Notch signalling in Drosophila.. BMC Dev Biol.

[50] Baladron V, Ruiz-Hidalgo MJ, Nueda ML, Diaz-Guerra MJ, Garcia-Ramirez JJ (2005). dlk acts as a negative regulator of Notch1 activation through interactions with specific EGF-like repeats.. Exp Cell Res.

[51] Mei B, Zhao L, Chen L, Sul HS (2002). Only the large soluble form of preadipocyte factor-1 (Pref-1), but not the small soluble and membrane forms, inhibits adipocyte differentiation: role of alternative splicing.. Biochem J.

[52] Wang Y, Zhao L, Smas C, Sul HS (2010). Pref-1 interacts with fibronectin to inhibit adipocyte differentiation.. Mol Cell Biol.

[53] Ferron SR, Charalambous M, Radford E, McEwen K, Wildner H (2011). Postnatal loss of Dlk1 imprinting in stem cells and niche astrocytes regulates neurogenesis.. Nature.

[54] Ahmed EI, Zehr JL, Schulz KM, Lorenz BH, DonCarlos LL (2008). Pubertal hormones modulate the addition of new cells to sexually dimorphic brain regions.. Nature neuroscience.

[55] Kokoeva MV, Yin H, Flier JS (2005). Neurogenesis in the hypothalamus of adult mice: potential role in energy balance.. Science.

