# Supplementary figures and images for: DLK1 Is a Somato-Dendritic Protein Expressed in Hypothalamic Arginine-Vasopressin and Oxytocin Neurons
Source: PLoS One. 2012 Apr 26;7(4):e36134. doi: 10.1371/journal.pone.0036134 (PMC3338567; doi:10.1371/journal.pone.0036134)

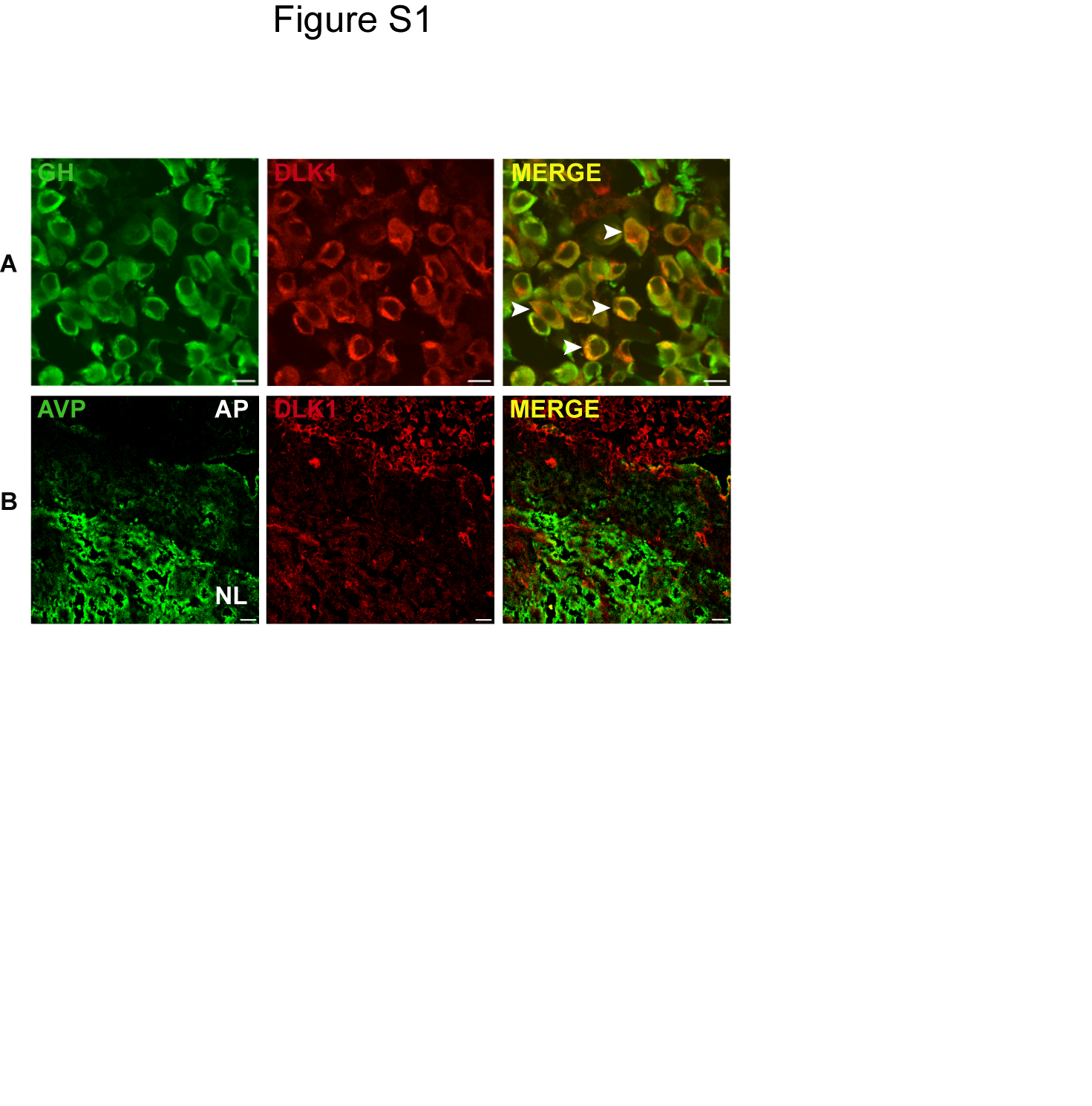

Supplement: Figure S1 — Dlk1 pituitary immunostaining. A) Pituitary immunostaining with antibodies to DLK1 (C-19) and growth hormone (GH). A confocal 0.6 µm-thick section obtained with a 63× objective. Primary antibody used for GH staining is the polyclonal guinea pig anti rat growth hormone (NIDDK, Torrance, CA). Arrow heads indicate double staining. B) DLK1 is not expressed in the neuronal lobe of the pituitary. Dual immunofluorescence staining on a single 0.6 µm-thick confocal section obtained using a 40× objective. Scale bars, 10 µm; AP: anterior pituitary; NL: neuronal lobe of the pituitary. (TIF) [file pone.0036134.s001.tif]

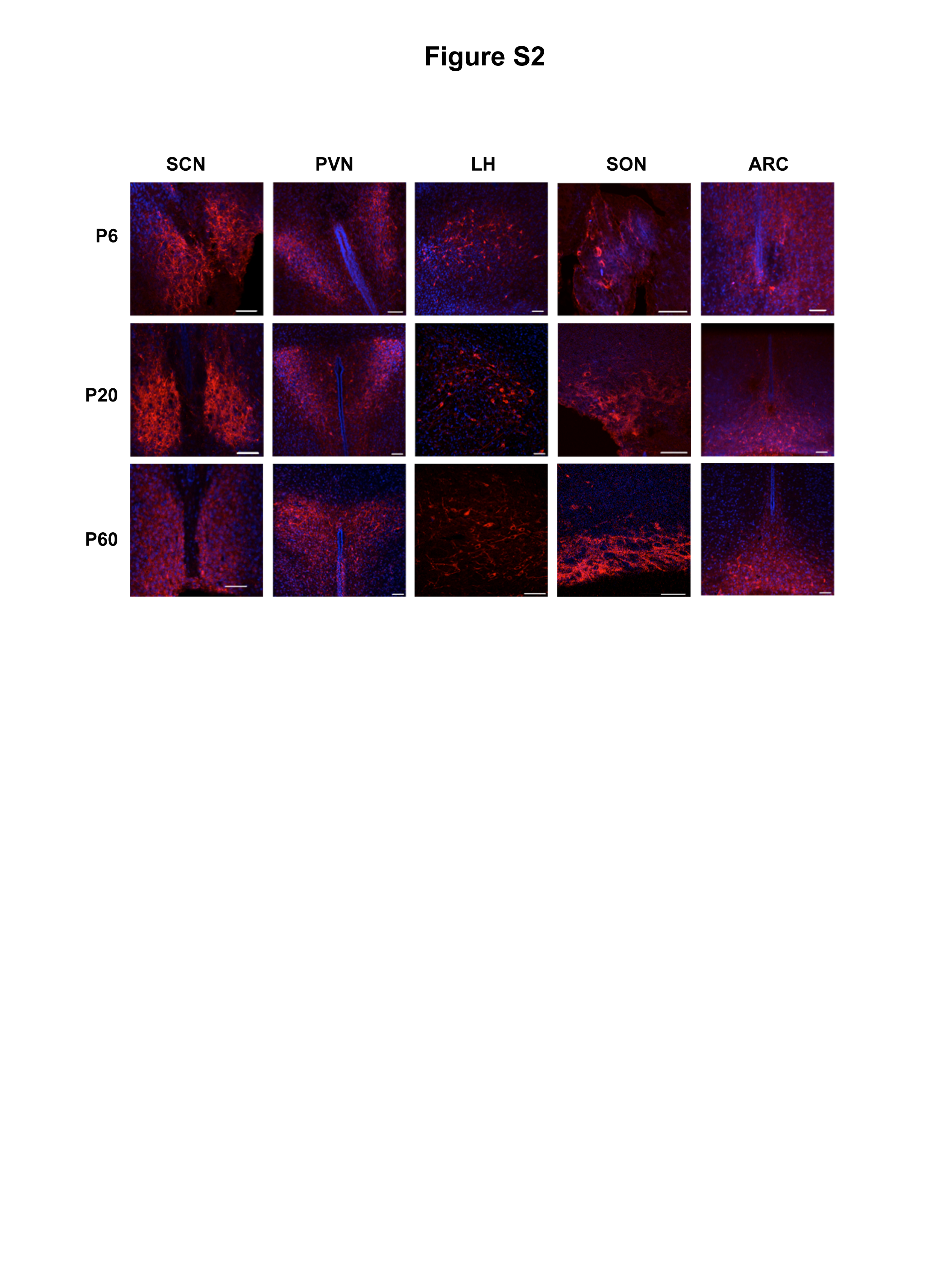

Supplement: Figure S2 — Hypothalamic nuclei expressing DLK1 do not change with age in male mice. Immunostaining with the C-19 antibody was performed on free-floating sections at P6, P20, and P60 as described in the “materials and methods" section. Confocal 0.6 µm-thick sections were obtained with a 40× objective. Suprachiasmatic nucleus (SCN), Paraventricular nucleus (PVN), lateral hypothalamic nucleus (LH), supraoptic nucleus (SON), and arcuate nucleus (ARC). Scale bars: 50 µm. The stained hypothalamic nuclei are the same at the different ages. To quantify a possible variation of DLK1 expression in each hypothalamic nucleus at the different stages, RT-qPCR of Dlk1 should be performed on dissected hypothalamic nuclei. (TIF) [file pone.0036134.s002.tif]
