# Supplementary material for: DLK1 Is a Somato-Dendritic Protein Expressed in Hypothalamic Arginine-Vasopressin and Oxytocin Neurons
Source: PLoS One. 2012 Apr 26;7(4):e36134. doi: 10.1371/journal.pone.0036134 (PMC3338567; doi:10.1371/journal.pone.0036134)
Supplement: Table S1 — PCR primers. Sequences of PCR primers used for polymerase-chain-reaction amplification (PCR) or quantitative real-time PCR (qPCR). (DOC) [file pone.0036134.s003.doc]

**Table S1: PCR primers**

|  | **Gene** | **Primer sequences** | **Anneal Temp.**  **(°C)** | **Reference** | **Product size (bp)** |
| --- | --- | --- | --- | --- | --- |
| **PCR** | **DLK1** | F5’-AGTACGAATGCTCCTGCACA | 56 | NM_0100524 | 1042 |
|  |  | R5’-GAGGAAGGGGTTCTTAGATAGCG |  |  |  |
|  | **GAPDH** | F5’-AATGTGTCCGTCGTGGATCTGA | 60 | NM_008024 | 83 |
|  |  | R5’-GATGCCTGCTTCACCACCTTCT |  |  |  |
| **qPCR** | **DLK1** | F5’-TGGCTGTGTCAATGGAGTCT | 60 | NM_0100524 | 121 |
|  |  | R5’-TTCTCCAGGTCCACGCAAGT |  |  |  |
|  | **KiSS-1** | F5’-TGGTGCAGCGGGAGAAGGA | 60 | NM_178260 | 136 |
|  |  | R5’-CTCCCTGCCTTGGCCTCTAC |  |  |  |
|  | **GAPDH** | F5’-AATGTGTCCGTCGTGGATCTGA | 60 | NM_008024 | 83 |
|  |  | R5’-GATGCCTGCTTCACCACCTTCT |  |  |  |
|  | **Jagged1** | F5’-CTGTAAAAACGGTGGCAGCTG | 60 | NM_013822 | 90 |
|  |  | R5’-TTGTTCTCACAGTGCGCTCC |  |  |  |
|  | **Jagged2** | F5’-ATGGATCTCTGTGAACCAAGC | 60 | NM_010588 | 100 |
|  |  | R5’-TTCTTGCCACCAAAGTCTTC |  |  |  |
|  | **Notch 1** | F5’-TGACTATCTCGGCGGCTTTT | 60 | NM_008714 | 84 |
|  |  | R5’-GACAGGCAGTCGTTGATCTCC |  |  |  |
|  | **Notch 2** | F5’-TGTCTGAACGAGAAGGTCCAG | 60 | NM_010928 | 92 |
|  |  | R5’-GGCATCGGAGACATACGTG |  |  |  |
|  | **Dll1** | F5’-CGGCTTCTATGGCAAGGTCT | 60 | NM_007865 | 102 |
|  |  | R5’-GTGTAGCCTCCGTCAGGGTTA |  |  |  |
|  | **Hey1** | F5’-TGCGCCTCCAAACTGTCTC | 60 | NM_010423 | 92 |
|  |  | R5’-GAAGGGCTCAGTAGGTGGAAG |  |  |  |
|  | **Hey2** | F5’-TACTTTGATGCCCATGCTCTT | 60 | BC_105652 | 96 |
|  |  | R5’-GCCTTCCACTGAGCTTAGGT |  |  |  |
|  | **Hes1** | F5’-CGAAGGGCAAGAATAAATGAA | 60 | BC_018375 | 81 |
|  |  | R5’-ATGCCGGGAGCTATCTTTC |  |  |  |
|  | **Hes5** | F5’-GATGCTCAGTCCCAAGGAGA | 60 | NM_010419 | 87 |
|  |  | R5’-TGCTCTATGCTGCTGTTGATG |  |  |  |
