# Supplementary material for: DLK1 Is a Somato-Dendritic Protein Expressed in Hypothalamic Arginine-Vasopressin and Oxytocin Neurons
Source: PLoS One. 2012 Apr 26;7(4):e36134. doi: 10.1371/journal.pone.0036134 (PMC3338567; doi:10.1371/journal.pone.0036134)
Supplement: Table S2 — Hypothalamic mRNA expression of Notch pathway genes on post-natal days (P) 6, 20 and 60. Total RNA from pooled hypothalami (n = 4) was reverse-transcribed and analysed using quantitative PCR as described in materials and methods. The mRNA levels are given relative to GAPDH mRNA levels. ns: not significant. (DOC) [file pone.0036134.s004.doc]

**Table S2: Hypothalamic mRNA expression of Notch pathway genes on post-natal days (P) 6, 20 and 60**

|  | | **Expression relative to GAPDH** | | | | | |
| --- | --- | --- | --- | --- | --- | --- | --- |
| **Genes** |  | **P6** | **P20** | **P60** | **P6vsP20**  ***p*** | **P6vsP60**  ***p*** | **P20vsP60**  ***p*** |
|  | **Jagged 1** | 0.3 ± 0.01 | 0.2 ± 0.02 | 0.3 ± 0.02 | <0.05 | ns | ns |
|  | **Dll1** | 0.2 ± 0.01 | 0.6 ± 0.03 | 0.5 ± 0.07 | <0.05 | <0.05 | ns |
|  | **Jagged 2** | 9.5 ± 0.4 | 7.3 ± 0.4 | 4.7 ± 0.2 | <0.05 | <0.01 | <0.05 |
|  | **Notch 1** | 7.2 ± 0.8 | 2.9 ± 0.04 | 5.2 ± 0.01 | <0,05 | ns | ns |
|  | **Notch 2** | 7.5 ± 0.3 | 5.9 ± 0.1 | 4.2 ± 0.5 | ns | <0.05 | ns |
|  | **Hes 1** | 3.8 ± 0.2 | 1.8 ± 0.01 | 3.3 ± 0.1 | <0.01 | ns | <0.05 |
|  | **Hes 5** | 26.7 ± 1 | 11.1 ± 0.3 | 7.0 ± 0.01 | <0.001 | <0.001 | <0.05 |
|  | **Hey 1** | 9.7 ± 0.01 | 13.2 ± 0.05 | 4.9 ± 0.01 | <0.001 | <0.001 | <0.001 |
|  | **Hey 2** | 10.4 ± 0.7 | 6.7 ± 0.2 | 8.2 ± 0.3 | <0.05 | ns | ns |
|  | **Dlk1** | 15.6 ± 1.9 | 38 ± 2.2 | 43.2 ± 2.4 | <0.001 | <0.001 | ns |
